# Current Bioinformatics resources in combating infectious diseases

**DOI:** 10.6026/97320630014031

**Published:** 2018-01-31

**Authors:** Amr T. M. Saeb

**Affiliations:** 1 Genetics and Biotechnology Department, Strategic Center for Diabetes Research, College of medicine, King Saud University, KSA

**Keywords:** Next-generation sequencing, pathogen identification, whole genome sequencing, genotyping, ribosomal (rRNA) gene, pathogenicity, virulence, resistome

## Abstract

Bioinformatics tools and techniques analyzing next-generation sequencing (NGS) data are increasingly used for the diagnosis and monitoring of infectious diseases. It is of interest to review the application of bioinformatics tools, commonly used databases and NGS data in clinical microbiology, focusing on molecular identification, genotypic, microbiome research, antimicrobial resistance analysis and detection of unknown disease-associated pathogens in clinical specimens. This review documents available bioinformatics resources and databases that are used by medical microbiology scientists and physicians to control emerging infectious pathogens.

## Background

The application of Bioinformatics tools and techniques in analyzing the increasing data generated in molecular biology, genomics, transcriptomics, and proteomics is gaining momentum [1]. Moreover, the amount of information gleaned in the form of databases and literature for generating molecular profiles and for collecting data related epidemiology of pathogens has been also mounting [2]. Therefore, the use of Bioinformatics tools and techniques in pathogen identification and typing, identifying markers for early diagnosis and treatment, enabling personalized interventions and predicting patient outcomes is imperative [3]. Bioinformatics aided next generation sequencing (NGS) data analysis are promising to identify clinically relevant viruses from a variety of specimen types [4]. Similarly, bacterial pathogens such as Francisella tularensis and Leptospira santarosai were successfully identified using culture-Independent NGS identification from primary human clinical specimens [5, 6]. The application of Bioinformatics techniques in the surveillance of pathogen outbreaks in fighting infectious diseases is also essential. Thus, this review documents available bioinformatics resources and databases that are used by medical microbiology scientists and physicians to control emerging infectious pathogens.

## Bioinformatics Tools for Pathogen identification and typing

Bioinformatics tools are extensively used in the identification, characterization, and typing of all kinds of pathogens. This followed the widespread use of genomic approaches in the diagnosis and management of viral, bacterial, and fungal infections. Applications of bioinformatics have been used in pathogen identification, detection of virulence factors, resistome analysis, and strain typing. Next-generation sequencing (NGS) technology supported by bioinformatics, phylogenetic, and patho-genomics analyses helped in the identification of the causative agent were a Clostridium haemolyticum isolate [3]. This isolate possesses virulence factors necessary to establish an infection and cause the all the observed symptoms. Thus, NGS holds considerable potential for pathogen identification isolated from human specimens using whole genome sequencing (WGS) assisted by powerful bioinformatics tools [7]. The application of Bioinformatics tools in analyzing WGS and Ribosomal (rRNA) gene sequencing data for the identification of both bacterial and fungal pathogens is becoming routine in recent years. The need for advanced yet improved bioinformatics tools in the analysis of NGS-rRNA sequencing data is emerging in microbiome studies [8]. The available bioinformatics tools used in sequence assembly & analysis and microbiome studies are given in Table 1.

The available Bioinformatics tool for microbiome studies does detection and removal of the amplification-derived chimeric sequence (Table 1). Most chimeras occur between sequences from closely related taxa. However, organisms from distant taxa also form chimeras. These could be classified as novel organisms if not properly identified as anomalous score. Thus, removal of chimeric sequences is an essential step in microbiome analysis. In addition to the above mentioned tools, there are automated pipelines dedicated for analyzing both processed data and raw sequences such as QIIME [9], Ribosomal Database Project (RDP) (http://rdp.cme.msu.edu/) [10], and mothur [11]. RDP contains sequence information of 3,356,809 bacterial 16S rRNAs and 125,525 fungal 28S rRNAs. RDB provides quality-controlled, aligned and annotated bacterial, archaeal 16S rRNA sequences, fungal 28S rRNA sequences and a suite of analysis tools to the scientific community. It contains a new Fungal 28S Aligner with updated Bacterial and Archaeal 16S Aligner. It also provides a pipeline for extended processing and analysis of high-throughput sequencing data, including single-strand and paired-end reads. Moreover, mothur is presently the highest cited bioinformatics tool for analyzing 16S rRNA gene sequences. Mothur enables to process data generated by different sequencing technologies such as, Sanger, PacBio, IonTorrent, 454, and Illumina (MiSeq/HiSeq).

Several comprehensive reference databases have been developed to facilitate accurate bacterial pathogen identification. The Greengenes database contain 1049116 aligned 16S rDNA records (http://greengenes.secondgenome.com/downloads) and SILVA contains 6,300,000 available SSU/LSU sequences of bacteria, archaea & eukarya (https://www.arb-silva.de/) and Human Oral Microbiome Database (HOMD) (http://www.homd.org) contains comprehensive information on the approximately 700- prokaryote species that are present in the human oral cavity. HOMD includes both static and dynamically updated annotations and bioinformatics analysis tools for both genomic sequences and processed 16S rRNA gene reference sequences for all the human oral microbes. MG-RAST server (http://metagenomics.anl.gov) is useful for WGS metagenomics analysis and it is more advanced compared with 16S rRNA sequencing [12]. MG-RAST server is an automated analysis platform for meta-genomes to present the quantitative understandings into microbial populations generated from sequencing data. The server provides options for upload, quality control, automated annotation and comparative analysis for shotgun and amplicon metagenomic samples as well as metatranscriptomes. Moreover, high-throughput sequencing (HTS) using Bioinformatics pipeline (ezVIR) was used to evaluate the entire spectrum of known human viruses and provided results that are easy to interpret and customizable. This pipeline works by identifying the most likely viruses present in the specimen using sequence data. The ezVIR pipeline generates strain typing reports, genome coverage histograms, and cross-contamination analysis for specimens prepared in series. This pipeline was able to identify DNA or RNA viruses in most collected clinical specimens. Tools are also available for the removal of host sequences from the NGS resulting pathogen and human sequence mixed pool. The filtering step is very important since the amount of viral sequencing in the resulting pool is usually less than 1%. For example, rapid identification of non-human sequences (RINS) (https://s3.amazonaws.com/changseq/kqu/) was able to precisely identify sequencing reads from non-human genomes in the used dataset and vigorously produces contigs from these sequences in less than two hours [4, 13]. The RINS is an intersection-based pathogen detection workflow that utilizes a user-reference genome set for the identification of non-human sequences in deep sequencing datasets. VirusSeq is an algorithmic method that is also used for detecting known viruses and their integration sites in the human genome using NGS data. VirusSeq was developed using PERL platform (http://odin.mdacc.tmc.edu/-xsu1/VirusSeq.html) [14]. HMMER3 compatible profile hidden Markov models (profile HMMs) were constructed within vFAM software (http://derisilab.ucsf.edu/software/vFam) to classify sequences as viral or non-viral [15]. PathSeq (http://www.broadinstitute.org/software/pathseq) was developed to identify both known and unknown microorganisms in NGS data.

NGS supported by bioinformatics tools has been used to catalog discrete organisms within complex yet poly-microbial specimens. Deep sequencing of 16S rRNA implies Actinomadura madurae causing mycetoma in diabetic patient [16]. However, conventional microbiological and molecular methods failed due to the overgrowth of Staphylococcus aureus. Later, the use of bioinformatics analysis in the identification of a bacterial pathogen was introduced elsewhere by Saeb et al. 2017 [3]. We have developed an analysis pipeline to identify and annotate the suggested pathogen. The quality of the reads was assessed and reads with score less than 20bp were removed. Secondly, the selected reads were subjected to Metaphlan software [17] for primary microbial identifications based on unique and cladespecific marker genes. BLAST program was used to map each read to the non-redundant nucleotide database of NCBI. Presence of high contamination with human non-pathogen sequences was observed. Later TMAP (https://github.com/iontorrent/TMAP) program was used to remove the contamination reads. The target non-human sequences were subjected to further analysis. MIRA software (version 4) [18] was used to perform de novo assembly for these non-human sequences. The selected sequences were mapped with bacterial genomes that were top ranked based on Metaphlan, BLAST findings. The pipeline used in the study was imported to the workflow system Tavaxy [3]. We further used QIIME pipeline for performing taxonomic assignment and for results visualizations [9]. Microbial typing is an important application in clinical microbiology, population genetics, and infection control [19, 20, 21]. The most commonly used techniques are Multilocus sequence typing (MLST), single locus sequence typing (SLST), multilocus variable-number of tandem repeats analysis (MLVA) and less commonly interspaced short palindromic repeats (CRISPR) [22, 23, 24]. Freely available databases for MLST data analysis, MLVA typing and SLST analysis are given in Table 2.

## Tools for Pathogenicity and virulence

An important bioinformatics tool to test the pathogenicity of a newly discovered bacterial pathogen is the PathogenFinder 1.1 (https://cge.cbs.dtu.dk/services/PathogenFinder/). PathogenFinder is a webserver used for the prediction of bacterial pathogenicity utilizing proteomic, genomic, or raw reads. The bacterial pathogenicity depends on groups of proteins known to be involved in pathogenicity [25]. This webserver utilizes a selection of proteins created without annotated function or known involvement in pathogenicity. It can predict pathogenicity for all taxonomic groups of bacteria with 88.6% accuracy. The approach of the program is not biased with known pathogenicity. Therefore the program could be used to discovery novel pathogenicity factors.

A recent method for predicting pathogenicity is the PaPrBaG (Pathogenicity Prediction for Bacterial Genomes) (https://github.com/crarlus/paprbag) based on machine learning and provided as R package [26]. PaPrBaG predicts pathogenicity by means of training on a large number of established pathogenic species in comparison with nonpathogenic bacteria. Suitable for NGS data with very low genomic coverages. PaPrBaG is a random forest based method for the assessment of the pathogenic potential of a set of reads belonging to a single genome. It helps in the prediction of novel, unknown bacterial pathogens. PaPrBaG provides prediction in contrast with other approaches that discard many sequencing reads based on the low similarity to known reference genomes.

Furthermore, the genomic contigs of a pathogen produced by NGS techniques are annotated using Prokaryotic Genomes Automatic Annotation Pipeline (PGAAP) available at NCBI. It can also be annotated using bacterial bioinformatics database and analysis resource (PATRIC) gene annotation service (https://www.patricbrc.org/app/Annotation) for pathogenicity and virulence factors. Virulence genes sequences and functions, corresponding to different major bacterial virulence factors of specific pathogen can also be collected from GenBank and validated using virulence factors of pathogenic bacteria database (http://www.mgc.ac.cn/VFs/), Victors, virulence factors search program (http://www.phidias.us/victors/) and PATRIC_VF tool (https://www.patricbrc.org/) [27]. However, in order to utilize all tools and links provided by PATRIC user should register in the main porter of the website.

## Bioinformatics tools for identifying and combating antimicrobial resistance

**Figure 1:**
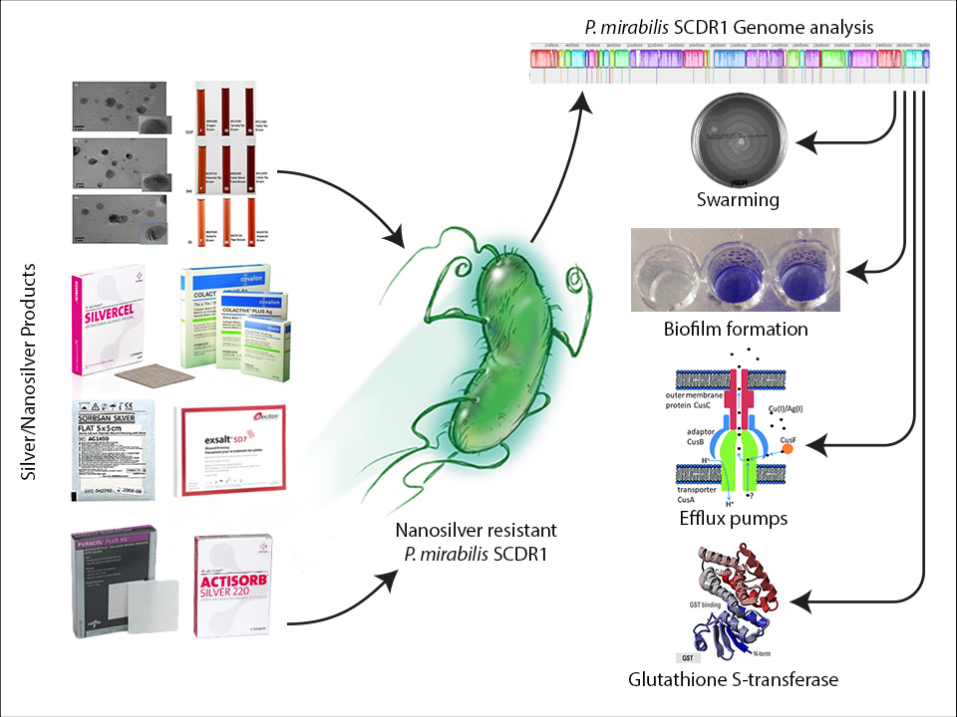
Resistome analysis of the first nanosilver resistance bacterium using the bioinformatics tools for identifying and combating anti-microbial resistance

The need for rapid, accurate detection and understanding of resistance factors and mechanisms are highly demanded in antimicrobial resistance. The genome contigs can be primarily investigated for the presence of antibiotic resistance loci using both PGAAP and PATRIC gene annotation services. Further, the presence of antibiotic resistance loci for the newly isolated bacterial pathogens can then be investigated using specialized search tools and services namely, Antibiotic Resistance Gene Search (https://www.patricbrc.org/), Genome Feature Finder (antibiotic resistance), ARDB (Antibiotic Resistance Genes Database) (https://ardb.cbcb.umd.edu/), CARD (The Comprehensive Antibiotic Resistance Database) (https://card.mcmaster.ca/), Specialty Gene Search and ResFinder 2.1 [27, 28, 29]. ResFinder 2.1 identifies acquired antimicrobial resistance genes and/or finds chromosomal mutations in total or partially sequenced isolates of bacteria. ResFinder is a web server that provides an appropriate way of identifying acquired antimicrobial resistance genes in completely sequenced isolates. It can be accessed at (www.genomicepidemiology.org). ResFinder is updated on new resistance genes regularly. Similarly, antibacterial biocide and metal resistance genes, can also be investigated using PGAAP, PATRIC gene annotation services, PATRIC Feature Finder searches tool and BacMet (antibacterial biocide and metal resistance genes database) (http://bacmet.biomedicine.gu.se/) [30, 31]. P.mirabilis SCDR1, the first Nanosilver resistant isolate contains pathogenicity and virulence factors to establish a successful infection. P.mirabilis SCDR1 contains several mechanisms for antibiotics and metals resistance including biofilm formation, swarming mobility, efflux systems, and enzymatic detoxification. P.mirabilis SCDR1 possesses several mechanisms that may lead to the observed Nanosilver resistance (Figure 1) [32].

## Conclusion

Several Bioinformatics tools are available for analyzing data for combating and control of infectious diseases as discussed in this review. However, there are several bioinformatics tools for drug resistance testing, pathogen-host interaction, infection and treatment outcomes. Nonetheless, the need to facilitate and incorporate bioinformatics tools and applications in clinical microbiology and infectious diseases through training of personnel and by developing simple yet robust user-friendly bioinformatics pipelines.

## Figures and Tables

**Table 1:** Bioinformatics tools for sequence assembly & analysis and microbiome studies

S. No	Tool Name	URL
1	Lasergene	http://dnastar.com
2	CLCbio workbench	http://www.clcbio.com/products/clc-main-workbench/
3	Geneious	http://www.geneious.com/
4	Mauve	http://gel.ahabs.wisc.edu/mauve
5	DECIPHER	http://DECIPHER.cee.wisc.edu
6	UCHIME algorithm	http://drive5.com/usearch/manual/uchime_algo.html
7	ChimeraSlayer	http://microbiomeutil.sourceforge.net/#A_CS
8	mothur	https://www.mothur.org/
9	AmpliconNoise	http://qiime.org/scripts/ampliconnoise.html
10	CATCh	http://science.sckcen.be/en/Institutes/EHS/MCB/MIC/Bioinformatics/CATCh

**Table 2:** Databases for MLST data analysis, MLVA typing and SLST analysis are listed

Databases for MLST data analysis			
S. No	Tool Name	URL	Information
1	Multi Locus Sequence Typing	http://www.mlst.net	MLST provides a portable, accurate, and highly discriminating typing system that can be used for most bacteria and some other organisms.
2	pubMLST	http://www.pubmlst.org	Public databases for molecular typing and microbial genome diversity.
3	Institut Pasteur MLST	http://www.pasteur.fr/mlst/	It hosts databases of multilocus sequence typing (MLST) and whole-genome based typing schemes, which are used for genotyping of bacterial isolates. They provide reference nomenclatures of microbial strains and are mainly intended for molecular epidemiology of pathogens of public health importance, detection of virulence and antimicrobial resistance genes, and for population biology research.
4	European Working Group for Legionella Infections (EWGLI) Sequence-based typing database	http://www.hpa-bioinformatics.org.uk/legionella/legionella_sbt/php/sbt_homepage.php	It aids in the investigation of outbreaks of legionellosis caused by L. pneumophila.
5	Environmental Research Institute, University College Cork	http://mlst.ucc.ie/	Contains 11614 of total records, 2389 Sequence types, 38 flaA alleles, 53 pilE allele, 72 asd alleles, 84 mip alleles, 96 mompS alleles, 54 proA alleles, 63 neuA alleles and 30 neuAh alleles)
Databases for MLVA typing			
6	MLVAbank	http://mlva.u-psud.fr/mlvav4/genotyping/	For genotyping of Acinetobacter baumannii, Bacillus anthracis, Brucella, Coxiella burnetii, Legionella pneumophila, Mycobacterium tuberculosis, Pseudomonas aeruginosa, Staphylococcus aureus and Yersinia pestis.
7	Groupe d'Etudes en Biologie Prospective	http://www.mlva.eu	For genotyping of Staphylococcus aureus, Streptococcus pneumoniae, Pseudomonas aeruginosa, M. tuberculosis, S. enterica and K. pneumoniae.
8	MLVA-NET	https://research.pasteur.fr/en/publication/mlva-net-a-standardised-web-database-for-bacterial-genotyping-and-surveillance/	It facilitates microbes genotyping for epidemiological purposes using polymorphic tandem repeat typing (MLVA), multiple locus sequence typing (MLST), single nucleotide polymorphisms (SNPs), and spoligotyping assays based upon clustered regularly interspersed palindromic repeats (CRISPRs).
9	Multiple-Locus Variable number tandem repeat Analysis	http://www.mlva.net/	Bordetella pertussis, Haemophilus influenzae, Neisseria meningitidis, Staphylococcus aureus and Streptococcus pneumoniae.
Databases for SLST analysis			
10	ccrB-typing tool	http://www.ccrbtyping.net/	
11	dru typing	http://dru-typing.org/site/	It contains 99 dru repeats and 531 dru types from 1 to 23 repeats as per 22nd of May 2017.
12	Ridom SpaServer	http://spaserver.ridom.de/	It aids in Surveillance of methicillin-resistant Staphylococcus aureus (MRSA). Single locus DNA-sequencing of the repeat region of the Staphylococcus protein a gene (spa) used for steadfast, precise and discriminatory typing of MRSA
13	CRISPRs web server	http://crispr.i2bc.paris-saclay.fr/	CRISPRcompar compares clustered regularly interspaced short palindromic repeats.
